# Error-dependent modulation of speech-induced auditory suppression for pitch-shifted voice feedback

**DOI:** 10.1186/1471-2202-12-54

**Published:** 2011-06-06

**Authors:** Roozbeh Behroozmand, Charles R Larson

**Affiliations:** 1 Speech Physiology Laboratory, Department of Communication Sciences and Disorders, Northwestern University, 2240 Campus Drive, Evanston, IL 60208, USA

## Abstract

**Background:**

The motor-driven predictions about expected sensory feedback (efference copies) have been proposed to play an important role in recognition of sensory consequences of self-produced motor actions. In the auditory system, this effect was suggested to result in suppression of sensory neural responses to self-produced voices that are predicted by the efference copies during vocal production in comparison with passive listening to the playback of the identical self-vocalizations. In the present study, event-related potentials (ERPs) were recorded in response to upward pitch shift stimuli (PSS) with five different magnitudes (0, +50, +100, +200 and +400 cents) at voice onset during active vocal production and passive listening to the playback.

**Results:**

Results indicated that the suppression of the N1 component during vocal production was largest for unaltered voice feedback (PSS: 0 cents), became smaller as the magnitude of PSS increased to 200 cents, and was almost completely eliminated in response to 400 cents stimuli.

**Conclusions:**

Findings of the present study suggest that the brain utilizes the motor predictions (efference copies) to determine the source of incoming stimuli and maximally suppresses the auditory responses to unaltered feedback of self-vocalizations. The reduction of suppression for 50, 100 and 200 cents and its elimination for 400 cents pitch-shifted voice auditory feedback support the idea that motor-driven suppression of voice feedback leads to distinctly different sensory neural processing of self vs. non-self vocalizations. This characteristic may enable the audio-vocal system to more effectively detect and correct for unexpected errors in the feedback of self-produced voice pitch compared with externally-generated sounds.

## Background

Surviving in an alien world is dependent upon the ability to distinguish between sensory inputs arising from self actions and those of others. The question of how the brain identifies self in an external environment has been discussed for decades. A well-accepted idea first introduced by von Helmholtz [1] and further expanded upon by von Holst and Sperry [2,3] in the visual system, suggested that the brain issues a copy of the motor commands (termed as efference copies) to predict sensory consequences of self-generated eye movements. The idea of sensory predictions based on efference copies has emerged as an important theoretical concept for sensory-motor integration in different modalities such as visual [4,5], auditory [6-8], and somatosensory [9,10] systems. According to this theory, the brain is capable of determining the source of sensory stimulation (self vs. non-self) by comparing efference copies of the motor predictions (corollary discharges) with the actual sensory stimuli. This process has been suggested to be mediated by subtractive comparison between corollary discharges and incoming feedback and has been proposed to result in motor-induced suppression (MIS) of self-generated sensory inputs that closely match the internal predictions.

The MIS of neural responses to self-generated sensory input has been suggested to result in different sensory neural processing of self vs. externally-generated feedback. In the somatosensory system, MIS was demonstrated by a reduction in the perceptual sensation of self-produced tactile stimulations compared with the same stimuli delivered by an external agent [10,11]. The early evidence for MIS in the auditory system comes from studies by Müller-Preuss et al. [12,13] in Squirrel monkeys in which it was shown that the electrical stimulation of vocal motor brain areas results in the reduction of activity in cortical auditory neurons. A similar effect was observed during voluntary sound production in crickets [14-16] and voluntary vocal production in Marmoset monkeys [6,17], showing that auditory neural responses to self-produced auditory feedback were suppressed during active sound production compared with passive listening to the playback of the same auditory stimuli. In humans, the N100 component of event-related potentials (ERPs) and its magnetoencephalographic (MEG) counterpart (M100) were shown to be suppressed in response to the onset of unaltered voice auditory feedback during active vocal production compared with passive listening [8,18,19]. Moreover, auditory neural responses to self-triggered (i.e. button press) tone stimuli were shown to be suppressed compared with those in response identical stimuli triggered by a computer [20-22].

A study by Heinks-Maldonado et al. [7] reported a similar suppression effect for N100 ERP responses to voice feedback during vocal production and demonstrated that the MIS of the N100 component at voice onset is largest for unaltered voice feedback and was reduced for conditions where voice feedback was pitch shifted (200 cents) or modified with an alien voice. Heinks-Maldonado et al. [7] suggested that N100 suppression was largest for unaltered voice feedback because motor predictions could maximally cancel the actual sensory input when the voice and its auditory feedback closely matched (i.e., no feedback error). This finding led to the hypothesis that identification of self-vocalizations, as measured by the suppression of N100 ERP responses, depends on the extent to which the acoustical parameters of voice feedback match the internal predictions about the intended vocal output, represented by the efference copies of motor commands during vocalization.

In order to test this hypothesis, it is necessary to specify the acoustical parameters that distinguish our own voices from those of others. Since voices can differ along many acoustical dimensions simultaneously (e.g. intensity, pitch, formants etc.), it is important to control the effect of all parameters while we investigate the effect of a single element in this identification process. As suggested by Heinks-Maldonado et al. [7], pitch frequency is possibly one of the factors that can help us distinguish between our own voices and those of other speakers. However, the acoustical parameters of the alien voices in their study [7] that led to the identification of self-vocalizations and consequently suppression of auditory neural responses to voice feedback during vocal production were not specified. There are several acoustical parameters (e.g., pitch, formants, etc) that can co-vary between one's own voice and those of others, and to understand the neural mechanisms involved in this process, it is necessary to learn which acoustical properties account for the suppression of self-voice.

In the present study, we tested the hypothesis that MIS of N100 responses to voice onset can be modulated by the degree of disparity between pitch frequencies of actual (sensory) and internally-predicted (efference copies) voice feedback. ERPs were recorded in response to five different magnitudes (0, 50, 100, 200 and 400 cents) of voice-onset pitch feedback perturbation during active vocalization and passive listening to the playback. The magnitude of pitch shift stimuli (PSS) was systematically increased to address the question how previously reported MIS of the N100 component is modulated for different levels of error in vocal pitch feedback. We predicted that the N100 suppression would be greatest for the unaltered voice feedback condition when there is no pitch disparity (error) between actual and intended voice output. However, when errors are introduced by pitch shifting the voice auditory feedback, MIS of N100 responses was predicted to be reduced during active vocalization compared with passive listening at voice onset.

## Results

**Figure 1:**
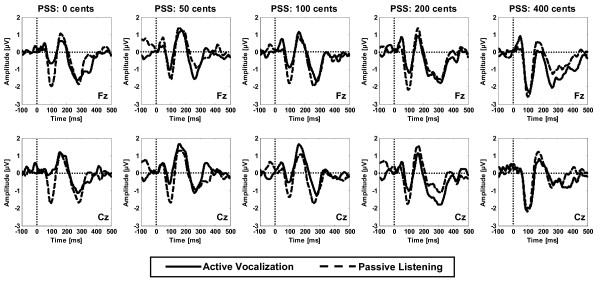
**Time course of the ERP responses to pitch-shifted voice feedback at voice onset for 0, 50, 100, 200 and 400 cents stimulus magnitudes**. ERPs responses from frontal (Fz) and central (Cz) EEG channels are overlaid for active vocalization (solid) and passive listening (dashed) conditions. The horizontal and vertical dashed lines in each subplot mark the baseline and stimulus onset, respectively.

**Figure 2:**
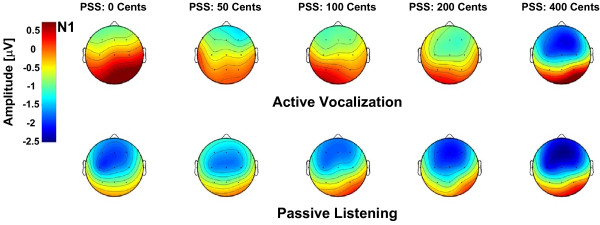
**Topographical scalp distributions of the N1 ERP component in response to 0, 50, 100, 200 and 400 cents pitch shifted voice feedback at vocal onset**. The top and bottom rows show the topographical maps of N1 distributions for active vocalization and passive listening conditions, respectively. The maps are calculated for 13 recording sites on the surface of the scalp (C_Z_, C_3_, C_4_, T_7_, T_8_, F_Z_, F_3_, F_4_, F_7_, F_8_, P_Z_, P_3_, P_4_).

SPSS (v.15.0, SPSS Inc.) was used to perform two-way (2 × 13) repeated-measures analysis of variances (Rm-ANOVAs) to separately analyze N1 responses (negativity around 100 ms) for different PSS magnitudes at voice onset with factors including condition (active vocalization vs. passive listening) and electrode position (Central: Cz, Left Centro-Medial: C_3_, Right Centro-Medial: C_4_, Left Temporal: T_7_, Right Temporal: T_8_, Fronto-Central: Fz, Left Fronto-Medial: F_3_, Right Fronto-Medial: F_4_, Left Fronto-Lateral: F_7_, Right Fronto-Lateral: F_8_, Parieto-Central: Pz, Left Parieto-Medial: P_3 _and Right Parieto-Medial: P_4_). The reported p-values associated with the statistical tests were corrected using Greenhouse-Geisser or Huynh-Feldt methods in conditions where the Mauchly's test indicated that the assumption of spherecity was violated (inhomogeneous variances). These methods correct for the violation of spherecity by choosing more stringent criteria for determining the degrees of freedom for the independent variables to ensure that the obtained F-values are valid. Results of the analysis for N1 revealed a significant main effect of condition for 0 (F(1,21) = 10.24, p = 0.004), 50 (F(1,21) = 4.98, p = 0.037), 100 (F(1,21) = 5.19, p = 0.033) and 200 (F(1,21) = 5.17, p = 0.034) cents PSS magnitudes but not for 400 cents PSS (F(1,21) = 2.76, p = 0.111). These results indicated that the N1 responses were significantly suppressed during active vocalization compared with passive listening only for pitch perturbations that were equal to or smaller than 200 cents but not for 400 cents perturbations (see Figure 1). A significant main effect of electrode position was also found for all tested PSS magnitudes including 0 (F(2.8,58.2) = 7.46, p = 0.000), 50(F(2.7,56.4) = 3.87, p = 0.017), 100 (F(2.3,48.2) = 4.50, p = 0.013), 200 (F(2.8,58) = 6.20, p = 0.001) and 400 (F(2.7,57.1) = 8.43, p = 0.000) cents. No significant condition × electrode position interaction was found for N1 responses to different PSS magnitudes, indicating that the scalp distribution of the N1 component was not significantly different during active vocalization compared with passive listening across PSS magnitudes (Figure 2).

The N1 responses were also separately analyzed for active vocalization and passive listening conditions using two-way (5 × 13) Rm-ANOVAs with factors including PSS magnitude (0, 50, 100, 200 and 400 cents) and electrode position. Results revealed significant main effects of PSS magnitude (F(2.8,58) = 5.03, p = 0.004), electrode position (F(2.3,47.3) = 3.25, p = 0.042) and a significant PSS magnitude × electrode position (F(7.6,159) = 3.12, p = 0.003) interaction for N1 responses during active vocalization. Post-hoc tests using Bonferroni's adjustment revealed that the significant main effect of PSS magnitude was due to significant differences between N1 responses for 400 vs. 0 (p = 0.011), 400 vs. 50 (p = 0.041), 400 vs. 100 cents (p = 0.038) and 400 vs. 200 cents (p = 0.049) stimulus magnitudes, indicating that 400 cents PSS elicited significantly larger (more negative or less suppressed) N1 responses compared with 0, 50, 100 and 200 cents stimuli at voice onset. The significant PSS magnitude × electrode position interaction indicated that the scalp distributions of N1 were different across stimulus magnitudes during active vocalization (see the top row head plots in Figure 2). For the passive listening condition, results revealed only a significant main effect of electrode position (F(3,63) = 12.46, p = 0.000). The absence of a PSS magnitude effect indicated that there were no systematic changes of N1 responses as a function of stimulus magnitude during passive listening. The absence of a PSS magnitude × electrode position interaction for passive listening indicated that there was no significant difference between scalp distributions of N1 across different stimulus magnitudes in this condition (see the bottom row head plots in Figure 2).

**Figure 3:**
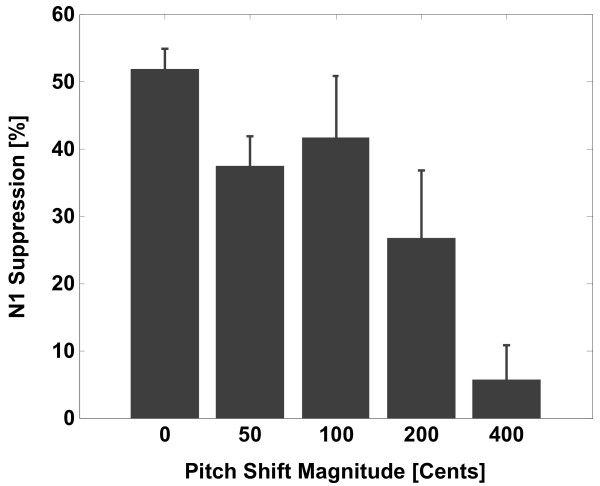
**The bar plot representation of the mean percentage of normalized N1 suppression for 0, +50, +100, +200 and +400 cents upward pitch shift stimulus (PSS) at voice onset**. The error bars show the standard error values for each stimulus magnitude, separately.

The normalized N1 suppression in percentage was calculated for each subject according to the following formula in which N1_LIS _and N1_VOC _are the peak amplitude of the N1 component during passive listening and active vocalization, respectively:

This normalized suppression index was first calculated across all 13 EEG channels for each subject and then grand averaged over all the subjects. The bar plots in Figure 3 show the mean percentage of suppression for normalized N1 amplitudes and the error bars represent the standard error values for different PSS magnitudes averaged over 22 subjects.

Results of the analysis for P2 potentials (positivity around 200 ms) using a two-way (2 × 13) Rm-ANOVA with condition and electrode position factors only revealed a significant main effect of electrode position for 0 (F(2.4,50.6) = 3.05, p = 0.047) and 50 (F(2.8,58.7) = 3.17, p = 0.034) cent pitch shift stimuli. P2 responses were also analyzed using two-way (5 × 13) Rm-ANOVAs for active vocalization and passive listening conditions separately with factors including PSS magnitude and electrode position. During active vocalization, results revealed only a significant PSS magnitude × electrode position interaction (F(8.6,180.3) = 1.99, p = 0.045). During passive listening, results of the analysis did not reveal any significant effect.

## Discussion

In the present study, the pitch perturbation paradigm was used to address the question of whether the extent of disparity between voice F0 output and its auditory feedback modulates motor-induced suppression of auditory neural responses at voice onset. Results of the analysis showed that the N1 ERP component was significantly suppressed during active vocalization compared with passive listening to unaltered (0 cents) and pitch-shifted voice feedback at 50, 100 and 200 cents stimulus magnitudes (Figure 1). However, when voice F0 feedback was shifted at 400 cents, the N1 suppression was almost completely eliminated. Also, the calculation of the normalized N1 suppression showed that the mean of normalized N1 suppression was largest (almost 52%) for unaltered voice feedback (0 cents shift) and decreased to 37%, 41%, 26% and 5% for pitch shift magnitudes of 50, 100, 200 and 400 cents, respectively (see bar plots in Figure 3).

Separate analysis of active vocalization and passive listening conditions revealed that the maximum N1 suppression for unaltered feedback and its reduction or elimination for pitch-shifted feedback resulted from a finding that during vocalization, the amplitude of N1 responses became larger (less suppressed) as the PSS magnitude increased whereas no such systematic changes of N1 responses occurred across PSS magnitude during passive listening. Our results indicated that 400 cents pitch-shifts elicited N1 responses that were significantly larger (more negative or less suppressed) than those elicited by 0, 50, 100 or 200 cents shifts during vocalization. However, no such a difference was observed for N1 responses to different stimulus magnitudes during passive listening. These findings indicate that the motor-induced suppression develops for small and moderately large disparities (up to 200 cents shift) between predicted (efference copies) and actual voice F0 feedback but not for very large shifts (e.g. 400 cents).

In addition, our results revealed a significant PSS magnitude × electrode position interaction only during active vocalization, indicating that the scalp distribution of N1 responses were different across stimulus magnitudes. As can be seen in Figure 2, N1 potentials have a prominent fronto-central distribution for 400 cents PSS magnitude that is different from those for other stimuli. This difference arises from larger (less suppressed) neural responses to the largest pitch error in voice feedback (400 cents) compared with smaller stimulus magnitudes during active vocalization, indicating that the motor-induced modulation of neural generators of N1 is different for larger compared with smaller disparities between vocal pitch output and its auditory feedback.

The findings of the present study are consistent with those of Heinks-Maldonado et al. [7] in which it was shown that the MIS of auditory neural responses at voice onset were greater for unaltered voice feedback and became smaller as feedback was pitch shifted or modified with an alien voice. In addition, results of our study expand upon the findings by Heinks-Maldonado et al. [7] by showing that MIS decreases or is even eliminated (400 cents shift) with increases in the magnitude of pitch perturbation in voice auditory feedback.

Other studies have suggested that, in addition to the acoustical parameters (e.g. pitch frequency), the disparity between spatial and temporal aspects of self-generated feedback with respect to efference copies can also modulate the neural processing of sensory input during execution of motor tasks. In the somatosensory system, perturbation in the trajectory and onset time of self-generated tactile stimulations were associated with an increase in the intensity of tickle sensation, indicating that unpredictable feedback was less suppressed by efference copies of motor commands [23]. In the auditory system, MIS of auditory responses was shown to develop only for conditions where there was no delay between the onset of motor actions such as button press [24] or vocalization [25] and the onset of auditory stimuli (temporal predictability). Similarly, auditory neural responses to self-triggered (button press) tones were shown to be maximally suppressed compared with passive listening for conditions where the frequency and onset time of stimuli were predictable, and the suppression was reduced if the frequency or onset time was unpredictable [26]. Consistently, results of the present study indicate that pitch predictability with relevance to efference copies for unaltered voice feedback results in a greater MIS of auditory responses compared with pitch-shifted feedback.

The MIS effects described above support the notion of an internal forward model for execution and monitoring of self-produced motor tasks. The forward model is suggested to incorporate efference copies of the motor commands that are used to make a comparison with actual sensory feedback [27]. This comparison examines the degree of disparity between spatial (e.g. trajectory), temporal (e.g. time delay) or acoustical (e.g. pitch) features of sensory feedback and the efference-based predictions that affect the processing of sensory neural information. This characteristic may enable the sensory-motor mechanisms to identify the source of sensory stimulations by monitoring the degree of feedback predictability via efference copies to distinguish between self- and externally-generated inputs. Our results suggest that the onset of unaltered voice feedback elicits N1 responses that are maximally suppressed by the efference copies of motor commands during vocal production. Suppression becomes less pronounced for moderate pitch disparities (e.g. 50, 100 or 200 cents shifts) and is almost completely eliminated for large pitch shifts (400 cents) in voice feedback. These findings indicate that the pitch frequency is possibly one of the important voice components in identification of self-voices during vocalization or speaking.

In addition to a role of MIS in identifying the source of feedback, suppression of auditory neural responses was suggested to play an important role in enhancing neural sensitivity for detecting unexpected changes (error) in self-voice feedback. A study by Eliades and Wang [28] showed that while Marmoset monkeys vocalized and received their own unaltered voice feedback, some cortical auditory neurons reduced their firing rate (suppression), but then significantly increased their activity in response to pitch-shifted voice feedback. However, other neurons that increased their discharge rate (excited) in responses to unaltered voice feedback during vocal production did not respond to pitch shifts in voice auditory feedback. These data suggest that during vocal production, MIS of some cortical auditory neurons by means of efference copies of motor commands may provide a mechanism to enhance their neural sensitivity for pitch error detection in the feedback of self-produced vocalizations. It has also been demonstrated in humans that when pitch shifts were presented after the onset of self-produced voice [25,29,30] or musical sounds [31], ERP responses were enhanced during active production of the motor task (e.g. vocalization or piano play) compared with when subjects passively listened to the playback of the same self-produced voices or music.

However, the extent of vocalization-induced enhancement was shown to be greater for 100 and 200 cents compared with 500 cents stimulus magnitude [32]. This latter effect was suggested to occur due to the fact that the motor act of vocalization increases neural sensitivity to detect F0 feedback perturbations in order to accurately detect and correct for vocal pitch errors during speaking. However, when feedback pitch was shifted at 500 cents, the vocalization-induced enhancement of ERPs was reduced, suggesting the system may have interpreted it as an external sound, and consequently became less sensitive. Therefore, identification of the source of auditory stimulation and systematic tuning of neural sensitivity based on the degree of disparity between voice F0 and its feedback may be important for vocal production because if the audio-vocal system was equally sensitive to pitch changes in self and externally-generated voices, variations in the pitch of environmental sounds or voices from different speakers could possibly lead to fluctuations in a person's voice during speaking.

Despite the fact that PSS magnitude is shown to modulate neural responses to voice feedback, it is still not clearly understood why ERPs (N1 component) are suppressed at voice onset [7,8] and, in contrast, are enhanced (predominantly P2 component) when the pitch shifts occur in the middle of vocalizations [29,32]. One possible explanation for the differential effect of stimulus onset time is that the reduction of N1 suppression at voice onset for larger PSS magnitudes reported previously [7] and in the present study may reflect mechanisms that enable the system to monitor and maintain an intended vocal output by subtractive comparison between actual voice feedback and internal representations provided by efference copies. Therefore, the unaltered auditory feedback from self-generated vocalizations that closely match the internally-represented feedback are more strongly suppressed at voice onset because they are fully predicted by the efference copies of motor commands. However, after voice onset, feedback-based monitoring of vocal output may rely on comparing the current state of incoming feedback with a representation that is continuously updated by feedback from previous vocalization states. Therefore, instead of suppression, the audio-vocal system becomes more sensitive and highly responsive when disparities emerge between the parameters of voice and its feedback in the middle of vocalization.

The above explanation suggests that the system performs at least two different functions, which require different mechanisms: one function of monitoring voice auditory feedback is to identify the source of voice feedback [7], and the second function is to correct for errors in production [33]. The first function takes place at the onset of vocalization, whereas the second function is activated after vocal onset and becomes important during vocalization [25]. While the details of these processes remain unknown, the suppression of cortical neural activity at vocal onset by means of motor-driven mechanisms may contribute to enhancing neural sensitivity for detecting pitch variations during vocal production. The brain may utilize the motor predictions to determine the source of incoming feedback in order to systematically decrease neural sensitivity to variations in the feedback of those voices that are not recognized as being self-generated. This proposal is supported by earlier findings in primates suggesting that there might possibly be a link between neural suppression and sensitivity enhancement to unexpected changes in voice F0 feedback [28]. Because the N100 component in the present study was most sensitive to feedback perturbations at vocal onset, and the P200 component is most sensitive to perturbations during vocalization [32], it is reasonable to suppose that these components represent the two different functions of the audio-vocal system, identification of self from external, and the monitoring of self vocalization.

With relevance to the neural processes of voice monitoring and control leading to MIS of auditory responses discussed above, a question remains as to what brain areas are involved in auditory feedback-based monitoring and control of vocal output during vocal production or speech. The anatomical organization of the audio-vocal mechanism has been widely studied using functional neuroimaging techniques in a variety of speech production and perception tasks. Results of these studies proposed an audio-vocal integration circuitry, including neural areas such as the superior temporal gyrus (STG), superior temporal sulcus (STS), planum temporale (PT), pre-motor cortex (PMC), inferior frontal gyrus (IFG), anterior insula [34,35] and the anterior cingulate cortex (ACC) [36] that may be involved in online monitoring and control of voice F0. Moreover, the bilateral increase in the activity of the superior temporal areas (mainly STG and STS) was reported in studies when human subjects received pitch-shifted feedback of their own voice compared with unaltered feedback during vocal production [37,38]. A similar effect of increased activity in superior temporal areas was also reported in conditions where feedback disparity was generated by introducing formant shifts [39] or voice-gated noise [40] in the auditory feedback during self-vocalization. The superior temporal activities were also shown to be significantly greater for passive listening to the playback of self-speech compared with when subjects actively produced them [40]. Results of the above neuroimaging studies are consistent with findings of the electrophysiological recordings in the present study that showed the increase in N1 activity (less suppression) for larger pitch errors (e.g. 400 vs. 0 cents) in voice feedback during active vocalization, leading to diminished MIS for larger pitch errors in voice feedback during vocalization compared with passive listening. These results suggest that the suppression of cortical auditory areas is likely to arise from neural mechanisms that utilize an internally-predicted representation (efference copies) of intended vocal output to monitor and control for feedback pitch error during active vocal production of speech sounds. Such a characteristic may be an important aspect of sensory-motor integration for distinguishing external (erroneous) from self-generated stimuli for maintaining the acoustical parameters of intended vocal output.

## Conclusions

The findings of the present study provide supporting evidence for the existence of an internal forward model during speech production. The results indicated that the motor-induced suppression of auditory neural responses is maximal for normal compared with pitch-shifted voice feedback. This effect suggests that the motor act of vocalization suppresses the sensory consequences of self-produced vocal output that is predicted by the efference copies of motor commands. However, when voice feedback is different from efference predictions, suppression is reduced or completely eliminated. This characteristic may represent the ability of the audio-vocal system to detect the source of incoming feedback in order to more effectively detect and control for vocal errors that occur in the feedback of self-produced voices compared with externally-generated feedback.

## Methods

### Subjects

Twenty two right-handed native speakers of American English (12 females and 10 males, 19-35 years of age) participated in this study. All subjects passed a bilateral pure-tone hearing screening test at 20 dB SPL (octave frequencies between 250-8000 Hz) and reported no history of neurological disorders or voice training. All study procedures, including recruitment, data acquisition and informed consent were approved by the Northwestern University institutional review board, and subjects were monetarily compensated for their participation.

### Stimulus and design

**Figure 4:**
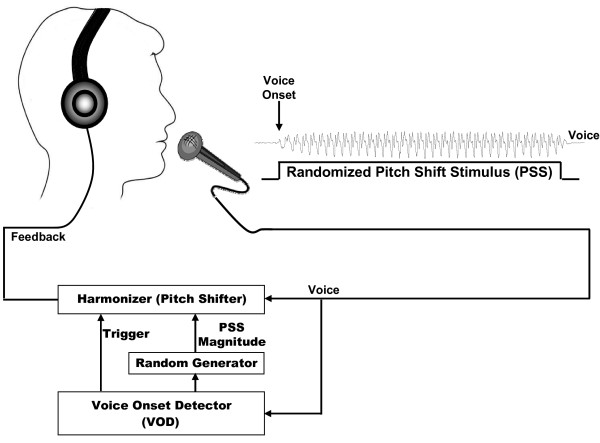
**Schematic of the experimental setup**. ERPs were obtained in response to randomly chosen 0, 50, 100, 200 and 400 cents pitch shifts in subjects' voice auditory feedback. The voice signal was fed to a voice onset detector (VOD) module that was used to detect voice onset. The VOD output cued a random generator function to randomly choose a stimulus magnitude and trigger the harmonizer to deliver pitch shifts at voice onset.

**Figure 5:**
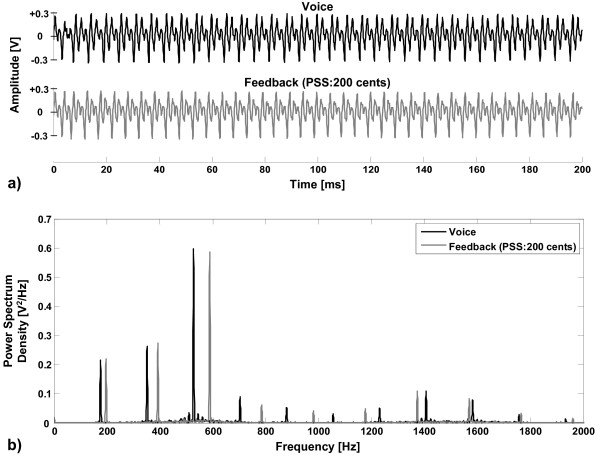
**An example of the a) sound pressure waveforms and b) frequency spectra of voice output for the vowel sound /a/ at pitch frequency of 175 Hz and its pitch-shifted (200 cents) auditory feedback (196 Hz)**.

The experiment consisted of three blocks of active vocalization each followed by one block of passive listening. During active vocalization, subjects were asked to sustain the vowel sound /a/ for approximately 1.5 seconds at their conversational pitch and loudness. This vocal task was repeated 200 times during each block while subjects took short breaks (2-3 seconds) between successive utterances. During each vocalization trial, subjects were presented with one of the randomly chosen upward pitch shift stimuli at 0, +50, +100, +200 and +400 cents magnitude starting from voice onset (Figure 4). Each active vocalization block was immediately followed by a passive listening block during which subjects passively listened to the playback of the same pitch shifts in the auditory feedback of their own voice. The total duration of each block was approximately 10-15 minutes and subjects kept their eyes open throughout the recording sessions to avoid alpha waves in the EEG signals. In total, 120 trials (3 × 200/5) during vocalization and 120 trials during passive listening were collected and analyzed for each PSS magnitude, separately. An example of the sound pressure waveforms and their frequency spectra for a vowel sound /a/ (pitch: 175 Hz) and its pitch-shifted (200 cents) auditory feedback (pitch: 196 Hz) are shown in Figure 5.

### Instrumentation

Subjects were seated in a sound-treated room in which their voice was picked up with an AKG boomset microphone (model C420) and amplified with a Mackie mixer (model 1202-VLZ3). The onset of the vocalization was detected using a voice onset detector (VOD) module implemented in Max/Msp (Cycling 74, v.5.0). The VOD output was used to trigger a function generator to randomly choose a PSS magnitude among 0, 50, 100, 200 and 400 cents (see Figure 4). The VOD output was also used to trigger an Eventide Eclipse Harmonizer to pitch shift voice feedback starting from voice onset. The pitch-shifted feedback lasted throughout the whole vocalization during each trial (1-1.5 sec). All parameters of the PSS such as duration, direction and magnitude were controlled by the Max/MSP. The Max/Msp also generated a TTL pulse to mark the onset of each PSS stimulus at voice onset for synchronized averaging of the recorded brain activity.

Voice, feedback and TTL pulses were sampled at 10 kHz using PowerLab A/D Converter (Model ML880, AD Instruments) and recorded on a laboratory computer utilizing Chart software (AD Instruments). Subjects maintained their conversational F0 levels with a voice loudness of about 70-75 dB, and the pitch-shifted feedback was delivered through Etymotic earphones (model ER1-14A) at about 80-85 dB. The 10 dB gain between voice and feedback channels was used to partially mask air-born and bone-conducted voice feedback. A Brüel & Kjær sound level meter (model 2250) along with a Brüel & Kjær prepolarized free-field microphone (model 4189) and a Zwislocki coupler were used to calibrate the gain between voice and feedback channels.

Following each active vocalization block, the recorded feedback channel was converted to a sound file to be played back during the passive listening condition. Two objective and one subjective method were used to calibrate the gain during the passive listening condition with respect to active vocalization. The objective methods included using the Brüel & Kjær sound level meter (model 2250) and a Zwislocki coupler to ensure the sound pressure level (dB-SPL) in the output of the insert earphones during passive listening was nearly identical to the earphone output level during vocalization. Furthermore, since the feedback channel was recorded on Chart recorder software (AD Instruments) during vocalization and listening, we verified that the voltage driving the earphones was identical during both conditions. Lastly, we asked subjects to verify that the sound intensity during vocalization and listening conditions was nearly identical.

### ERP acquisition and analysis

The electroencephalogram (EEG) signals were recorded from 13 sites on the subject's scalp (C_Z_, C_3_, C_4_, T_7_, T_8_, F_Z_, F_3_, F_4_, F_7_, F_8_, P_Z_, P_3_, P_4_) using an Ag-AgCl electrode EEG cap (10-20 system). Scalp-recorded brain potentials were amplified with a gain of 10 K (Grass amplifiers P511), low-pass filtered at 2 KHz (anti-aliasing filter) and then sampled at 10 kHz (PowerLab A/D Converter) and recorded using Chart software (AD Instrument). All amplifiers were calibrated according to the instructions from the manufacturers. All recorded EEG channels were referenced to linked earlobes and their impedances were maintained below 5 kΩ (Grass impedance meter EZM-5AB).

MATLAB (Mathworks, Inc.) software was used to analyze the recorded EEG signals to obtain ERPs in response to pitch-shifted feedback at voice onset. Data analysis was carried out by offline filtering of the recorded EEGs from all channels using a band-pass filter with cut-off frequencies set to 1 and 30 Hz (-12dB/oct). The filtered EEGs were then segmented into epochs ranging from -100 ms before and 500 ms after the onset of the PSS. Following segmentation, artifact rejection was carried out by excluding epochs with amplitudes exceeding +/-50 μV. Individual epochs were then subjected to baseline correction by removing the mean amplitude of the 100 ms-long pre-stimulus time window for each individual EEG channel. The minimum number of 100 epochs was averaged to calculate ERP responses for each stimulus magnitude at voice onset. The data were then grand averaged over 22 subjects for each stimulus magnitude, separately. For each individual subject, the peak amplitudes of N1 ERP components were extracted by finding the most prominent peaks in 20 ms-long time windows centered at 100 ms. This time window was selected based upon visual inspection of the grand averaged responses.

### Topographical Distribution Maps

The surface distribution maps of measures of brain activity in response to voice pitch feedback perturbation were created using MATLAB by mapping neural peak amplitudes of the ERPs for 13 electrode sites (C_Z_, C_3_, C_4_, T_7_, T_8_, F_Z_, F_3_, F_4_, F_7_, F_8_, P_Z_, P_3_, P_4_) over the surface of the scalp. These topographical distribution maps of neural activity were created by color coding the amplitudes of the ERP components using the interpolation method between adjacent electrodes to obtain a map of electrical activity distribution.

## Authors' contributions

RB and CRL conceived of the study and participated in its design. RB wrote the computer programs, coordinated the study, recruited participants, collected and analyzed the data. Both authors contributed to writing the manuscript and approved the final draft.
